# Decoding INPP5D/SHIP1: A Pivotal Regulator of Microglial Homeostasis in Alzheimer’s Disease

**DOI:** 10.1002/alz70859_103174

**Published:** 2025-12-25

**Authors:** Adrian L Oblak

**Affiliations:** ^1^ Indiana University School of Medicine, Indianapolis, IN USA

## Abstract

**Background:**

Alzheimer’s disease (AD) remains a devastating neurodegenerative disorder with complex pathophysiology, necessitating innovative therapeutic strategies. Among the emerging targets in AD research, INPP5D, a gene that encodes the SHIP1 phosphatase, stands out as a critical regulator of microglial function and a promising avenue for intervention. Microglia, the brain's resident immune cells, play a dual role in neuroprotection and neurodegeneration, contingent on various activation states. Recent findings by Oblak et al. have illuminated INPP5D’s central role in maintaining microglial homeostasis, positioning it as a key molecular regulator influencing the balance between beneficial and detrimental microglial responses.

**Method:**

SHIP1 exerts its effects as a negative regulator of intracellular signaling cascades, which in turn govern microglial processes such as phagocytosis, inflammatory cytokine release, and synaptic pruning. Dysregulation of these pathways has been linked to exacerbated neuroinflammation and impaired clearance of amyloid‐beta and other pathological substrates in AD. Leveraging insights from high‐throughput assays, advanced bioinformatics, and in vivo models, Oblak et al. have elucidated the mechanistic underpinnings of SHIP1’s influence on microglial activity and its downstream impact on neuronal health. These studies also underscore the potential of targeting SHIP1 to recalibrate microglial phenotypes, offering a novel therapeutic strategy for slowing or preventing AD progression.

**Result:**

In this presentation, we will discuss how the integration of functional genomics, structural biology, and pharmacological screening within the TREAT‐AD consortium has advanced our understanding of INPP5D/SHIP1. Focus will be placed on the discovery and characterization of small molecules that modulate SHIP1 activity and siRNAs that downregulate INPP5D expression. These innovative tools not only validate the therapeutic potential of INPP5D/SHIP1 but also pave the way for combination therapies and precision medicine approaches tailored to individual patients.

**Conclusion:**

The session aims to catalyze collaboration by presenting INPP5D/SHIP1 research as a cornerstone of TREAT‐AD’s mission to accelerate the discovery and development of AD therapies.

